# Predictors of discharge to home/community following inpatient-rehabilitation in a US national sample of Guillain-Barre-Syndrome patients

**DOI:** 10.1371/journal.pone.0286296

**Published:** 2023-05-25

**Authors:** David S. Kushner, Doug Johnson-Greene, Elizabeth R. Felix, Cheryl Miller, Maite K. Cordero, Stacy A. Thomashaw

**Affiliations:** 1 Department of Physical Medicine and Rehabilitation, University of Miami Miller School of Medicine, Miami, Florida, United States of America; 2 Therapy Operations, Encompass Health Corporation, Birmingham, Alabama, United States of America; St Paul’s Hospital Millennium Medical College, ETHIOPIA

## Abstract

**Background:**

Guillain-Barre-Syndrome (GBS), an autoimmune polyneuropathy causing acute flaccid paralysis, is a rare condition with1-2 cases per 100,000 annually (approximately 5000 cases/year) in the United States (US). There is a paucity of published data regarding patient outcomes in association with discharge destinations following inpatient-rehabilitation (IR) in this patient population, thus this study.

**Objectives:**

To analyze IR efficacy, and possible predictors of discharge to home/community in a US-national-sample of GBS patients.

**Methods:**

Retrospective-observational-cohort study of 1304 GBS patients admitted to IR comparing discharge disposition destinations (community/home, skilled-nursing-facility [SNF], or return to acute-care) by demographic (age, gender) and clinical variables (length-of-stay [LOS], case-mix-index [CMI], and Functional-Independence-Measure [FIM] score changes). Multinomial-logistic-regression and discriminant-function-analysis were performed to determine model fit in predicting discharge destination.

**Results:**

81.8% were discharged to home/community- average LOS 19-days, total-FIM-gain 43.2; 9.8% discharged to SNFs- average LOS 27.5-days, total-FIM-gain 27.2; and 8.4% discharged to acute-care- average LOS 15.4-days and total-FIM-gain 16.5, (F = 176, p < .001). Stepwise-linear-regression for prediction of community discharge showed change in FIM-Bed/chair/wheelchair-Transfers was the most significant predictor (Wald = 42.2; p < .001), followed by CMI (Wald = 26.9; p < .001), change in FIM-walking/wheelchair (Wald = 14.9; p < .001), and age (Wald = 9.5; p < .002). Using discriminant-function-analysis to test model validity for predicting discharge disposition, FIM-change for Bed/chair/wheelchair Transfers, Walking, and Self-Care as predictors resulted in a classification rate of 78.1%, 92% of variance explained, and Eigenvalue of .53 (p < .001).

**Conclusions:**

Total-FIM scores improved in all groups, and most patients were discharged to home/community suggesting IR efficacy. The ability to transfer bed/chair/wheelchair was the most important predictive factor associated with discharge destination.

## Introduction

Guillain-Barre-Syndrome (GBS), an acute inflammatory demyelinating polyneuropathy, is the most common cause of nontraumatic acute flaccid tetraplegia worldwide, having an incidence of 1.2–3 per 100,000 population per year, accounting for approximately 5,000 new cases annually in the United States [1,2]. Usual symptoms/signs include areflexia, dysautonomia, and weakness with progression to flaccid quadriparesis. GBS is a monophasic illness, however some patients can deteriorate after first stabilizing or improving on treatment with intravenous immunoglobulin (IVIG) and/or plasma exchange [3,4]. Prognosis is usually good with early detection, prompt treatment, and rehabilitation.

Approximately 40% of patients who are hospitalized with GBS will require admission to inpatient rehabilitation (IR) for multidisciplinary treatment following acute care [2,5], particularly if there was need for respiratory support or prolonged treatment in an intensive care unit (ICU). A recent study showed that IR likely improves functional outcomes in GBS beyond the contribution of expected neurological recovery alone [6]. Approximately 35% of GBS patients are older than 50 years [4]. Comparison of IR improvements in mobility and self-care in older patients with neurologic conditions showed GBS having the best functional improvement compared to stroke, multiple sclerosis, and Parkinson disease at time of discharge [7]. Older patients may be at greater risk to develop respiratory failure with need for assisted ventilation in intensive care units [8]. Up to 40% of GBS patients may need assisted ventilation, and a proportion of these patients may have dysautonomia, dysphagia, and develop ventilator associated pneumonia, sepsis, pneumothorax, and prolonged ICU stays [1,8]. The average length-of-stay (LOS) reported in the medical literature for GBS patients requiring transfer to IR is approximately 34 days in acute care [9]. Multidisciplinary IR treatment is necessary to hasten functional recovery for severe cases of GBS following acute care.

There is limited data regarding functional outcomes and discharge disposition destinations in GBS patients following IR. Studies of GBS patient functional outcomes after IR have been limited by a lack of multi-center rehabilitation collaborations and the ability to aggregate data across hospital systems. Collaborative studies involving traumatic brain injury and spinal cord injury have yielded advancements that characterize these populations and improve comprehensive care, treatment outcomes, and quality of life [10,11]. The focus of much research in GBS has been on patient care in the acute phase and improving survival instead of long-term disability, and quality of life. The goal of this study was to assess IR efficacy, and to determine predictors associated with discharge to home/community in patients with GBS following IR using data from a national rehabilitation hospital system in the United States (US). Based on the current literature, we predicted that discharge to home/community would be associated with greater Functional Independence Measure (FIM) gain in IR for bed transfers, walking, and self-care and would be inversely associated with age, illness complexity, and length of stay.

### Methods

This was a retrospective observational cohort study of GBS patients admitted to IR over 3 years, January 1^st^, 2017, to December 31^st^, 2019, at a national rehabilitation hospital system in the United States and Puerto Rico. There were no age limits, but a primary diagnosis of GBS that was diagnosed and treated during acute care prior to admission to IR was required for study inclusion. Diagnosis of GBS was made by licensed physicians in the acute care hospital setting prior to inpatient rehabilitation. The International Classification of Diseases, Tenth Revision (ICD-10) is a national coding system used by physicians and other healthcare providers to classify and code all diagnoses, symptoms, and procedures recorded in conjunction with hospital care in the United States. Patients admitted to IR with a primary diagnosis of GBS made during acute care were coded using the ICD-10 code for Guillain-Barré syndrome, G61.0. Data in this study were selected from among all persons who had an admitting diagnosis to IR if GBS was established in the acute care hospital and having the ICD-10 Code for Guillain-Barré syndrome, G61.0.

All patients were included in this study who had an admitting diagnosis of GBS, who had documented FIM scores for both admission and discharge, and for whom there was a documented discharge disposition destination to either home/community, back to acute care, or to a skilled nursing facility. No participants were excluded from the study who fulfilled these inclusion criteria. Criteria for admission to IR included the ability to participate in three hours or more therapy per day, medical stability, and the clinical judgment of the admitting rehabilitation attending physician regarding the potential benefits of the program to the patient. Time of discharge was decided by the multidisciplinary rehabilitation team and was considered when patients reached their expected functional goals. Multidisciplinary therapies, including physical, occupational, and speech therapies, focused on individualized functional goals that were identified on admission to IR by the treatment team. Patients were targeted to receive three hours of therapy each day. This study was approved by the national IR hospital system’s institutional corporate clinical research review/ethics board, known as the *Encompass Health Clinical Research Review Committee*.

### Measures

Demographic data were collected on the following variables: age and gender. Outcome variables included IR LOS, Functional Independence Measure (FIM) scores for admission and discharge to IR, and discharge disposition location (home/community, Skilled-Nursing-Facility (SNF), or back to an acute care hospital). Because of its proven validity and reliability, and its ability to measure functional capacity as it relates to burden of care, the FIM was used to assess functional outcome [12]. All FIM data were collected using the protocol established by the Uniform Data System for Medical Rehabilitation (UDSMR) in Buffalo, New York [12,13]. FIM scores collected on admission and discharge to IR included total-FIM scores (range 18–126) for an overall summary of a patient’s functional ability. Specific FIM scores collected on admission and discharge to IR included Walk/Wheelchair (ability to walk and/or operate a wheelchair, range 1–7), Bed/Chair/Wheelchair Transfers (ability to transfer between bed, chair or wheelchair, range 1–7), and Self-Care (range 6–42); these measures were used to gain insight regarding a patient’s capacity to perform these activities important to functional independence. Self -care FIM scores included component scores of ability to self-feed, groom, bathe, dress upper body, dress lower body, and perform toileting hygiene. Higher FIM scores indicate better functional performance. All the national IR facilities had successfully completed the UDS reliability certification process before collection of data used in the study. FIM LOS efficiency scores were also used as measures in this study, and these scores were calculated as FIM score change (discharge FIM score minus admission FIM score) divided by LOS.

Case-mix-index (CMI) scores from UDSMR were also compared in this study as a measure of clinical severity. The CMI scores are used by the Centers for Medicare and Medicaid Services (CMS) to determine hospital reimbursement rates for Medicare and Medicaid beneficiaries in the United States [14,15]. CMI is an indirect measure calculated on discharge of the severity of a patient treated at an IR facility and includes factors such as age, functional status, and LOS. A higher CMI score suggests a more medically/clinically complicated patient, while a lower CMI score suggests a less medically/clinically complicated patient.

### Statistical analyses

Age, FIM scores, CMI scores, and length-of-stay (LOS) were compared by discharge disposition location (home, SNF, or acute-care) using one-way analysis of variance (ANOVA). Gender distribution across discharge disposition location was tested using chi-square. Stepwise linear regression was performed, with discharge disposition as the outcome variable, and gender, age, LOS, CMI, and change in FIM scores (discharge score minus admission score for each of Walk/Wheelchair, Bed/Chair/Wheelchair Transfers, and Self-Care scores) as predictor variables. Possible FIM score ranges are summarized above under *Measures*. Finally, discriminant function (canonical discriminant function) analyses were computed to determine significant predictors for discharge outcomes (i.e., group membership), as well as to determine the overall classification rate (i.e., accuracy of the model). IBM SPSS Statistics version 25 was used for all analyses with p< .05 as the level of significance.

## Results

This study included 1304 GBS patients, ages 4–93 years, admitted to a national IR system from January 1, 2017, to December 31, 2019. The population of patients included in this study represents the largest group of GBS patients currently available in a multicenter study at this time. Significant differences were found among the three discharge disposition destinations for age, LOS, and CMI (see Table 1, all p < 0.001), but no significant differences were found for gender (χ2 = 0.45, p = 0.8). Posthoc Scheffe’ analyses to control for multiple comparisons revealed significant differences for nearly all comparisons, suggesting that main effects were not based on a single significant comparison and that the variables of age, LOS and CMI by discharge destination were quite different between each of the three discharge destination groups. Descriptive characteristics by discharge disposition can be found in Table 1.

**Table 1:** Demographic/Descriptive results summary and analysis of variance by discharge destination (n = 1304).

VARIABLE	ACUTE N = 109	SNF N = 128	COMMUNITY N = 1067	Significance
**Age**	64.4 (SD = 15.7) [range = 5–89 years]	57.2 (SD = 17.9) [range = 19–93 years]	56.1 (SD = 17.2) [range = 4–93 years]	F = 13.66; p< .001
**Gender** **(% male)**	54%	51%	53.4%	F = 1.54; p = .82
**Length of Stay**	15.4 (SD = 23.8)	27.5 (SD = 11.6)	19.0 (SD = 13.9)	F = 23.4; p < .001
**Case Mix Index (CMI)**	2.41 (SD = 0.76)	2.6 (SD = 0.66)	1.93 (SD = 0.85)	F = 50.9; p < .001

Note: CMI = case mix index; SNF = skilled nursing facility.

Overall, 81.8% of the study sample were discharged to home/community, with an average LOS of 19 days; 9.8% of the study sample were discharged to SNFs, with an average LOS of 27.5 days; and 8.4% of the sample were discharged back to acute-care hospitals, with an average LOS of 15.4 days. FIM gains were highest among those discharged to the community with an average of 43.2 compared to SNF FIM gain of 27.2 and acute care FIM gain of 16.5.

Consistent with our hypothesis community discharge was associated with younger age (p < .001), shorter LOS (p < .001), CMI scores suggesting less medical complexity (p < .001), and greater FIM gains for walking (p < .001), bed-transfers (p < .001), and self-care (p < .001). Community discharges averaged 3.1 (1.9) FIM-point gains per IR day compared to 1.1 (0.7) for SNF, and 1.6 (2.0) for acute care (F = 92.4 p < .001). Table 2 summarizes FIM gain by discharge destination.

**Table 2:** Functional independence measure (FIM) status summary and analysis of variance by discharge destination.

Variable	ACUTE N = 109	SNF N = 128	COMMUNITY N = 1067	Significance
**Admit Walk/WC**	1.5 (SD = 1.1)	1.5 (SD = 1.1)	1.8 (SD = 1.3)	F = 5.18 p = .006
**Discharge Walk/WC**	2.6 (SD = 2.0)	4.0 (SD = 2.0)	5.5 (SD = 1.3)	F = 220.19 p < .001
**Admit Bed Transfers**	1.4 (SD = 0.80)	1.1 (SD = 0.40)	2.0 (SD = 1.2)	F = 44.1 p < .001
**Discharge Bed Transfers**	2.2 (SD = 1.5)	2.6 (SD = 1.6)	5.3 (SD = 1.5)	F = 352.3; p < .001
**Admit Self-Care**	13.1 (SD = 6.3)	11.1 (SD = 5.2)	17.3 (SD = 7.2)	F = 58.7 p < .001
**Discharge Self-Care**	19.1 (SD = 8.9)	21.8 (SD = 8.8)	34.3 (SD = 8.1)	F = 273.3 p < .001
**Admit Motor FIM**	23.3 (SD = 10.2)	20.4 (SD = 8.9)	31.5 (SD = 13.3)	F = 59.3 p < .001
**Admit FIM Total**	47.7 (SD = 13.7)	41.9 (SD = 10.8)	58.7 (SD = 16.6)	F = 80.6 p < .001
**Discharge FIM Total**	64.2 (SD = 19.5)	69.1 (SD = 18.8)	101.9 (SD = 18.5)	F = 347.3 p < .001
**LOS Efficiency**	1.6 (SD = 2.0)	SD = 0.7)	3.1 (SD = 1.9)	F = 92.4 p < .001

WC = Wheelchair; FIM = Functional Independence Measure; SD = Standard Deviation; SNF = Skilled Nursing Facility.

Model building using stepwise linear regression was used to predict community discharge. Consistent with our hypotheses, statistical analyses revealed that change in Bed/Chair/Wheelchair Transfers FIM was the most significant predictor (Wald = 42.17; p < .001), followed by CMI (Wald = 26.9; p < .001), change in FIM-Walking/wheelchair (Wald = 14.9; p < .001), and age (Wald = 9.5; p < .002) (Table 3). Age, LOS efficiency, and change in Bed/Chair/Wheelchair Transfers FIM were all significant for prediction of discharge to SNFs (Table 3). Prediction of SNF discharge showed that LOS efficiency was the most important predictor (Wald = 38.3; p < .001). Stepwise linear regression to predict discharge back to acute care showed that change in walking/wheelchair FIM was the most important predictor (Wald = 23.14; p < .001) (Table 3).

**Table 3:** Multilinear-logistic-regressions to predict patient discharge destination.

Variable		B	S.E.	Wald	df	Sig.	Exp(B)
**Community**	Age	-.017	.005	9.472	1	.002*	.983
	LOS Eff	.174	.078	4.910	1	.027*	1.190
	CMI	-.629	.121	26.927	1	.000*	.533
	Walk WC FIM Change	.198	.051	14.872	1	.000*	1.219
	Bed Transfer FIM Change	.520	.080	42.172	1	.000*	1.682
	Self-Care FIM Change	.033	.018	3.499	1	.061	1.034
	Constant	1.285	.456	7.948	1	.005*	3.614
**Skilled Nursing Facility (SNF)**	Age	.032	.007	21.279	1	.000*	1.033
	LOS Eff	-.977	.158	38.321	1	.000*	.376
	CMI	.227	.147	2.385	1	.123	1.254
	Change FIM Walk WC	.091	.062	2.148	1	.143	1.095
	Bed Transfer FIM Change	-.372	.094	15.521	1	.000*	.690
	Self-Care FIM Change	.048	.021	5.267	1	.022*	1.049
	Constant	-3.014	.600	25.254	1	.000*	.049
**Acute Care**	Age	-.003	.007	.172	1	.679	.997
	LOS Eff	.209	.075	7.843	1	.005*	1.233
	CMI	.409	.147	7.747	1	.005*	1.505
	Change FIM Walk WC	-.346	.072	23.135	1	.000*	.708
	Bed Transfer FIM Change	-.532	.121	19.465	1	.000*	.587
	Self-Care FIM Change	-.086	.025	12.112	1	.001*	.918
	Constant	-.855	.541	2.492	1	.114	.425

LOS Eff = length of stay efficiency; CMI = case mix index; FIM = functional independence measure score; WC = Wheelchair.

Using discriminant function analysis to test model validity for prediction of discharge to the community, FIM-change for Bed Transfers, Walking, and Self-Care as predictors resulted in a classification rate of 78.1%, 92% of the variance explained, and an Eigenvalue of .53 (p < .001).

## Discussion

To our knowledge, this is the first study to evaluate IR efficacy, and the potential role of age, gender, change in FIM scores, medical complexity (CMI scores), and LOS in association with discharge destination following IR in a US national multi-center population of patients with GBS. All discharge destination groups showed improvement in total-FIM scores suggesting IR efficacy. The overwhelming majority of GBS patients in this study 81.8% were discharged to home/community, versus 9.8% that were discharged to SNFs, and 8.4% that were transferred back to acute care hospital wards also suggesting efficacy of IR at least in facilitating home/community discharges within an average LOS of 19 days. The average IR LOS for GBS in this study was shorter than those previously reported in the medical literature at approximately 26 days [9]. A United Kingdom (UK) national study of functional outcomes and efficiency of in-hospital rehabilitation in a national cohort of patients with inflammatory polyneuropathies that included 118 GBS patients found IR cost effective and efficient, albeit with an average LOS of 72 days [16]. A recent systematic review of randomized controlled trials also suggests efficacy of rehabilitation interventions including IR for GBS patients [17]. Our study showed that gender was the only factor that did not have a significant association with discharge disposition. Analyses in this study to predict community discharge revealed that change in FIM-bed/chair/wheelchair transfers was the most significant predictor, followed by CMI, change in FIM-walking/wheelchair, and age.

This study demonstrated an association between change in FIM scores and discharge disposition locations following GBS IR, which is consistent with our hypotheses. Although all discharge groups demonstrated an improvement in total-FIM scores by discharge, as expected lesser gains were noted in the patient groups discharged back to acute care and to SNFs. Intuitively, there would be lesser gains in FIM scores in the group of GBS patients that were discharged back to acute care due to the concomitant shorter LOS time frame in IR for functional improvement in mobility and ADLs due to medical complications. As expected, stepwise linear regression to predict discharge back to acute care showed that change in the walking/wheelchair FIM score was the most important predictor since these patients being transferred back to acute care have a shorter LOS in IR, with less time to improve in function (Table 3). Also as expected, LOS efficiency was the most important predictor of discharge to a SNF which was not surprising because these GBS patients do not make functional gains quickly. Similarly, greater functional dependence for mobility and ADLs and lesser gains in FIM scores would be expected in the group of GBS patients discharged to SNFs. Interestingly, a patient’s ability to transfer from bed to chair was the most important predictive factor associated with discharge disposition location, followed by the ability to ambulate and/or operate a wheelchair (Tables 2 and 3). Thus, in addition to working with patients on these functional tasks, IR treatment should also include practical education of family/caretakers on safe transfers prior to discharge, which may increase the likelihood of discharge to the community.

In this study, discharges from IR back to acute care occurred on average at about 2 weeks, to home/community at about 3 weeks, and to SNFs at about 4 weeks. Causes for acute care transfers during GBS IR may include medical complications such as GBS relapses, venous thromboembolism/pulmonary embolism, falls and associated injuries, infections, and uncontrolled comorbid conditions. Up to 5% of GBS patients may have relapses after first stabilizing or improving on therapy [3,18], and these patients are among those at risk for transfers from IR back to acute care units or hospitals. Findings in our study differed from the medical literature, with only 8% transfers back to acute care versus estimates as high as 40% of GBS patients requiring transfers between acute care units or hospitals with clinical deterioration, and up to 30% requiring more than one acute care transfer [9].

The obvious reason for the more prolonged LOS for those GBS patients that were discharged to SNFs in this study was the more limited capacity for functional improvement in this group compared to those discharged to home/community. As mentioned above in this discussion, a UK study demonstrated that patients having inflammatory polyneuropathies that included GBS may need more prolonged in hospital rehabilitation care to recover functional independence to return to home/community [16]. Circumstances that may have contributed to the acute care transfers and SNF discharges that are unknown in our study include premorbid factors such as co-morbid chronic medical conditions, and the various factors associated with the initial preadmission acute care GBS hospitalization/treatment such as prior treatment in an intensive care unit, intubation/mechanical ventilation, plasmapheresis, intravenous human immunoglobulin antibody administration, medical complications, and the need for endotracheal tubes and/or percutaneous gastrostomy tubes. Also, as expected, average CMI scores suggest greater medical complexity in those patients that were discharged to SNFs or back to acute care than those discharged to home/community (Tables 1 and 3). Further research is needed to clarify the interacting role of specific factors during GBS IR which may contribute to acute care transfers and SNF discharges that were not directly assessed in this study.

## Study limitations

Unknown factors in this study which may have affected functional outcomes and discharge destinations may include: 1. Specific patient co-morbidities/chronic conditions, 2. Patients’ prior functional status including abilities to perform transfers, ambulation, and selfcare prior to GBS, 3. Specific treatments that patients received for GBS during acute care prior to IR, and 5. Complications of GBS during acute care prior to IR admission such as secondary pneumonia infections, sepsis, or secondary critical illness myopathy weakness. Also unknown in this study were specific reasons for the transfers back to acute care. Furthermore, although this study seems to suggest efficacy of the IR care on GBS patient functional outcomes, there is no way to know with certainty whether the observed recovery was due to the natural course of the disease since a control group was not used for this comparison. Interestingly however, a recently published study found that the performance of daily tasks improved during IR for Guillain-Barré syndrome, beyond the improvement expected after the neurological motor recovery, and remained high after discharge, suggesting a likely contribution of rehabilitation to the functional outcome [6].

## Conclusions

This study seems to suggest that GBS IR treatment is efficacious since all groups demonstrated an improvement in total-FIM scores by discharge, and most patients (82%) were discharged to home/community following IR. Regarding the data presented here, a patient’s ability to transfer from bed to chair seems to be the most important predictive factor in determining discharge disposition location, followed by the ability to ambulate and/or operate a wheelchair. GBS IR treatment should especially target these functional tasks in patients and include practical education of family/caretakers on safe transfers prior to discharge which may increase likelihood of a community discharge. Further research is necessary to investigate other factors (such as those mentioned in the limitations section) which may also affect discharge disposition location in GBS patients following IR.

## Supporting information

S1 File (XLSX)
